# Live attenuated RHΔ*tkl1* and PruΔ*pp2a-c* mutants of *Toxoplasma gondii* are promising vaccine candidates conferring protection in pigs

**DOI:** 10.1186/s40249-026-01451-8

**Published:** 2026-05-06

**Authors:** Ze-Xuan Wu, Yu Kang, Zhi Zheng, Wen-Bo Hao, Shi-Bo Huang, Yu-Xuan Wang, Xiao-Nan Zheng, Xing-Quan Zhu

**Affiliations:** Shanxi Key Laboratory of Animal Disease Research, Prevention and Control, College of Veterinary Medicine, Shanxi Agricultural University, Taigu, Jinzhong, 030801 Shanxi Province People’s Republic of China

**Keywords:** *Toxoplasma gondii*, RHΔ*tkl1* and PruΔ*pp2a-c* mutants, Pig, Live attenuated vaccine, Immunization

## Abstract

**Background:**

*Toxoplasma gondii* is an apicomplexan parasite which infects nearly all warm-blooded animals and humans, causing zoonotic toxoplasmosis. Pork infected with *T. gondii* is considered a significant source of human infection. Currently, no commercial vaccines are available for porcine toxoplasmosis globally, thus a safe and effective vaccine is urgently needed. This study evaluated the immuno-protective effects of two *T. gondii* gene knockout attenuated strains, RHΔ*tkl1* and PruΔ*pp2a-c*, in pigs.

**Methods:**

Pigs immunized by intramuscular injection with 1 × 10^7^ tachyzoites of attenuated RHΔ*tkl1* or PruΔ*pp2a-c* strains were challenged orally with 1.000 sporulated oocysts of the Pru strain at 28 days post-vaccination (dpv), followed by a secondary challenge via intraperitoneal injection of 1 × 10^7^ Pru strain tachyzoites at 70 dpv. Clinical signs were monitored. *T. gondii*-specific antibody levels were examined by enzyme-linked immunosorbent assay. The protective efficacy was evaluated by analyzing pathological lesions, pathological score, parasite load, brain cyst burden, and by mouse bioassay. GraphPad Prism software was employed to perform the log-rank (Mantel Cox) test, Student’s *t* test and one-way ANOVA.

**Results:**

Pigs immunized with either RHΔ*tkl1* or PruΔ*pp2a-c* exhibited only low-grade fever. Combined with pathological changes and pathological scores, these findings support the moderate safety of both strains. Pigs immunized with either RHΔ*tkl1* or PruΔ*pp2a-c* and subsequently challenged with *T. gondii* Pru oocysts and tachyzoites exhibited a sharp increase in *T. gondii*-specific antibodies, which remained high for 5 weeks (*P* < 0.01). Pathological lesions were alleviated after immunization, with a significant reduction in parasite load observed in tissues including the brain, heart, gastrocnemius, longissimus dorsi, psoas major, and diaphragm (*P* < 0.01). Furthermore, a marked decrease in brain cyst burden was recorded (*P* < 0.0001). Mouse bioassay results confirmed a significant reduction in the proportion of mouse brain tissues positive for *T. gondii* genomic DNA in the group immunized and then challenged, compared to the non-immunized challenged group (*P* < 0.0001).

**Conclusions:**

The two live attenuated RHΔ*tkl1* and PruΔ*pp2a-c* mutants of demonstrated moderate safety, immunogenicity, and protective efficacy in pigs, identifying them as potential candidate vaccines against porcine toxoplasmosis.

**Graphical Abstract:**

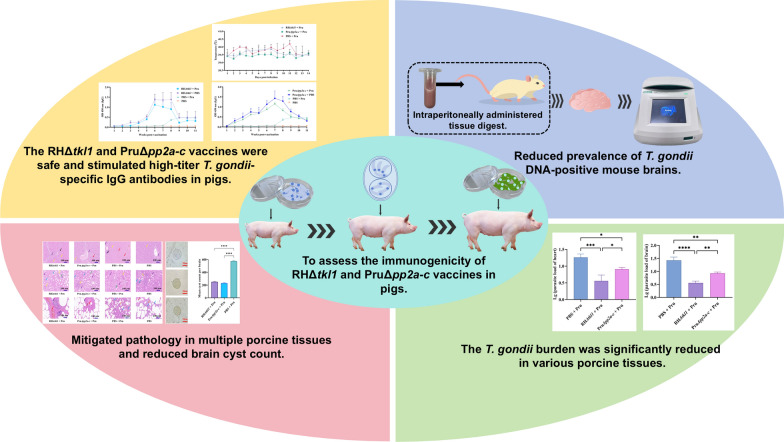

**Supplementary Information:**

The online version contains supplementary material available at 10.1186/s40249-026-01451-8.

## Background

*Toxoplasma gondii* is a protozoan parasite that can infect all warm-blooded animals, including humans and livestock [1]. Approximately one-third of the world human population has been infected by *T. gondii* [2]. In healthy people, *T. gondii* infection is usually asymptomatic without obvious ill effects. However, primary infections in immunosuppressed individuals can cause acute cerebral or systemic diseases, and the reactivation of latent tissue cysts can cause severe or even deadly consequences [3]. Pregnant women infection with *T. gondii* can cause miscarriage, stillbirth, or congenital anomalies [4, 5]. Epidemiological evidence indicates that undercooked meat, especially pork, is a major source of foodborne transmission for human infection with *T. gondii* [6, 7]. Tissue cyst in pork can persist for more than 2 years [8]. Thus, to reduce the risk of human infection with *T. gondii*, it is necessary to suppress the cyst formation in pigs. One of the ideal prevention and control strategies is to develop effective and safe vaccine against porcine toxoplasmosis [9, 10].

*T. gondii* has a complex life cycle involving multiple hosts [11]. The diversity of *T. gondii* strains and their successful evasion of immune surveillance through multifaceted manipulation of the host immune system present major challenges to the development of an effective vaccine [12, 13]. To date, the only commercial vaccine is the attenuated tachyzoite S48 strain (Toxovax^®^) commercially applicable to sheep. This vaccine reduces abortion and neonatal mortality in sheep while improving the birth weight of lambs [14]. However, the reliance on mammalian cell culture for tachyzoite growth results in a very short shelf life and high production costs [15]. Moreover, no vaccines against *T. gondii* are currently licensed for use in pigs. A number of experimental vaccine approaches have been tried, including the use of S48 strain tachyzoites, crude *T. gondii* rhoptry proteins plus Quil-A, *T. gondii* rROP2 plus the Iscomatrix adjuvant, dense granule proteins GRA1 and GRA7, *T. gondii* lysate antigens, excreted and secreted antigens, and Pru*Δcdpk2* [16–21]. These vaccines can significantly reduce, but not completely prevent, the formation of tissue cysts.

Advances in studies of vaccines against *T. gondii* have shown that live attenuated vaccines are the most promising option [22]. With the wide use of the Clustered Regularly Interspaced Short Palindromic Repeats-CRISPR Associated Protein 9 (CRISPR-Cas9) technology, generating gene deletion mutants as live attenuated vaccine candidates has provided a promising novel approach for controlling toxoplasmosis [23]. Several gene-deleted attenuated strains confer effective protection against *T. gondii* infection in mice [24–29]. The protein phosphatase 2A-C (PP2A-C) subunit is one of the three core components comprising the *T. gondii* PP2A holoenzyme, serving as its catalytic subunit [30, 31]. Previous studies have shown that PP2A-dependent dephosphorylation events play essential roles in the virulence, pathogenesis and stage conversion of *T. gondii* [31, 32]. Mice infected with the PruΔ*pp2a-c* strain exhibited reduced mortality and no detectable brain cysts [31]. Our previous study had demonstrated that the PruΔ*pp2a-c* mutant can induce strong protective efficacy against *T. gondii* infection in mice and cats [33]. *T. gondii* tyrosine kinase-like 1 (*tkl1*) is an essential virulence factor in *Toxoplasma* [34]. Parasites lacking *tkl1* have defects in host cell attachment, resulting in impaired growth in vitro and a complete virulence attenuation in mice [34]. Vaccination of RHΔ*tkl1* tachyzoites provokes significant protection against acute, chronic, and congenital toxoplasmosis in mice [35].

The two live attenuated RHΔ*tkl1* and PruΔ*pp2a-c* mutants of *T. gondii* confer robust immuno-protection against *T. gondii* infection in mice. However, it remains unclear whether they can elicit a protective immune response in pigs. Therefore, in this study, we investigated the protective immunity conferred by immunization with the attenuated *T. gondii* knockout strains RHΔ*tkl1* and PruΔ*pp2a-c* against pig toxoplasmosis, and further assessed their potential as candidate vaccines against pig toxoplasmosis.

## Methods

### Animals and parasites

Twenty-four Large White/Landrace cross bred pigs, aged 8 weeks, including females and castrated males, were randomly allocated in cages (four animals each). The animals were left to acclimatize for 7 days before the experiment. For the duration of the experimental period, a standard commercial porcine feed was provided to all animals, and water was available ad libitum. Eight-weeks-old female Porton mice (a minimally inbred strain) were purchased from Beijing Sibeifu Biotechnology Co. Ltd. [16]. All mice were fed under specific pathogen-free conditions at 50%–60% humidity and 22 °C, and provided with adequate food and water ad libitum. Mice were acclimatized for 1 week prior to the commencement of the experimental studies. Animal experiments were conducted following the principles of minimizing animal suffering and protecting animal welfare as much as possible. The tachyzoites of *T. gondii* Type II Pru strain, and the constructed gene knockout RHΔ*tkl1* and PruΔ*pp2a-c* strains were replicated in human foreskin fibroblast (HFF) cells, maintained in DMEM containing 2% fetal bovine serum (FBS), and cultured in an incubator at 37 °C with 5% CO_2_ [31, 35]. The infectious oocysts of *T. gondii* Pru strain were maintained at the Laboratory of Parasitic Diseases, College of Veterinary Medicine, Shanxi Agricultural University [33]. The maintenance and care of experimental animals complied with the Institutional Animal Care and Use Committee of Shanxi Agricultural University (Approval No.: SXAU-EAW-2021XM121001).

### Pig vaccination and challenge

**Fig. 1:**
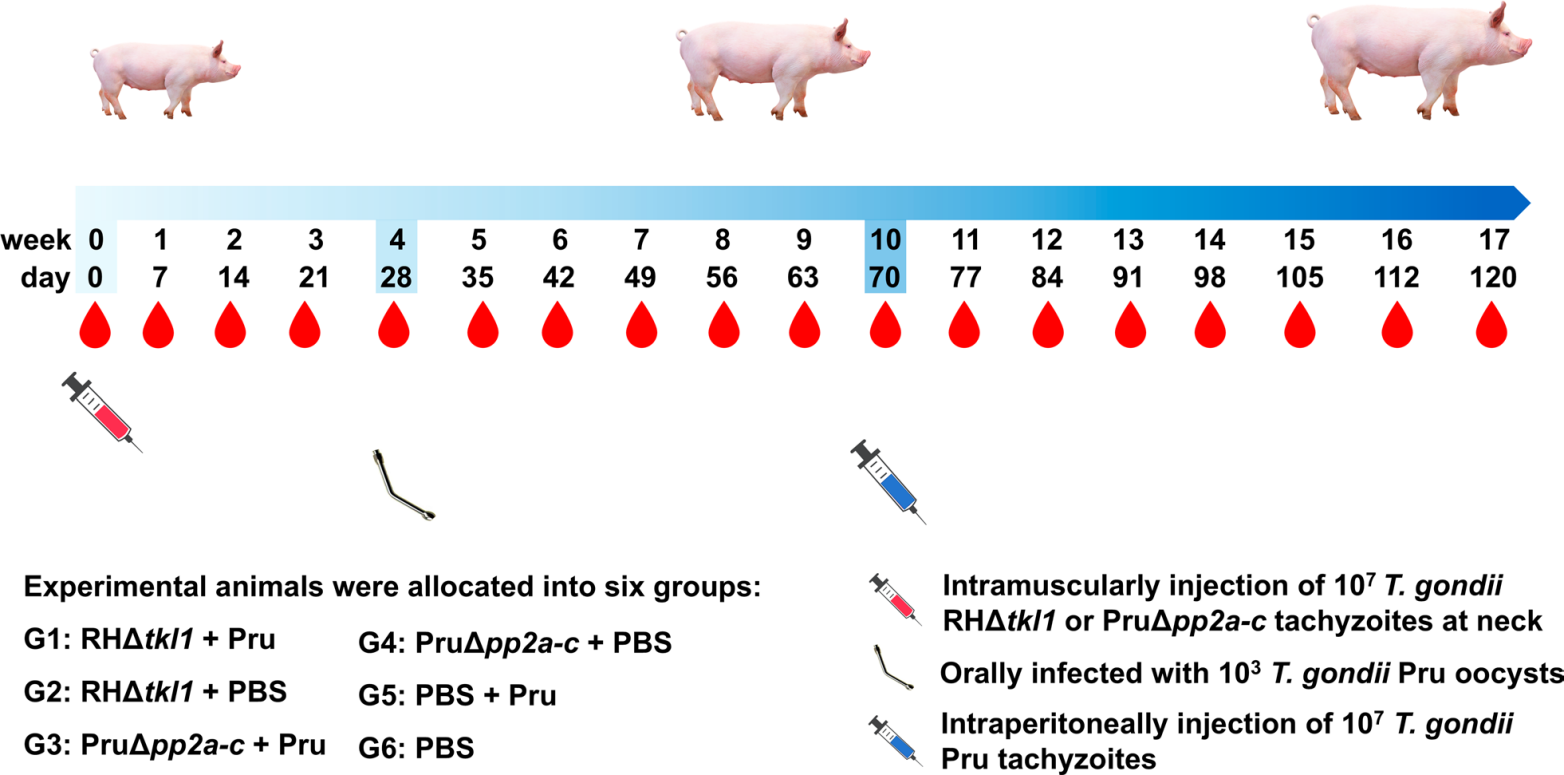
Scheme of the vaccination and challenge experiment. Experiment days are indicated as numbers (0–120)

The 24 pigs were divided into six groups (G) depending on experimental immunization and challenge infection (see Table 1 and Fig. 1). Twenty-eight days post-vaccination (dpv), animals in G1, G3 and G5 were orally challenged with 10^3^ *T. gondii* oocysts of the Pru strain. On 70 dpv, animals in groups G1, G3 and G5 were challenged intraperitoneally with 1 × 10^7^ tachyzoites of the *T. gondii* Pru strain. Figure 1 shows the timeline of the experiment.

**Table 1:** Animal groupings for vaccination with live attenuated strains of *Toxoplasma gondii* and challenge infection

Group	Vaccination and/or challenge		
	Day 0	Day 28	Day 70
1 (*n* = 4)	1 × 10^7^ RHΔ*tkl1* tachyzoites	1,000 Pru oocysts	1 × 10^7^ Pru tachyzoites
2 (*n* = 4)	1 × 10^7^ RHΔ*tkl1* tachyzoites	PBS	PBS
3 (*n* = 4)	1 × 10^7^ PruΔ*pp2a-c* tachyzoites	1,000 Pru oocysts	1 × 10^7^ Pru tachyzoites
4 (*n* = 4)	1 × 10^7^ PruΔ*pp2a-c* tachyzoites	PBS	PBS
5 (*n* = 4)	1 mL PBS	1,000 Pru oocysts	1 × 10^7^ Pru tachyzoites
6 (*n* = 4)	1 mL PBS	PBS	PBS

### Sampling

Rectal temperatures of all pigs were examined daily for 14 days post-immunization or post-infection. Blood sampling was carried out weekly from days 0 to 119 of the experiment, stored at 37 ℃ for 2 h, and then centrifuged at 3,000 × *g* for 10 min. The separated serum was stored at −20 ℃ until further analysis. All 24 pigs were euthanized at day 120 by electrical stunning followed by severing of the jugular vein and exsanguination. Tissues (brain, spleen, liver, kidney, lung, hilar lymph nodes, chop, loin, left tricep, left semitedinosus, heart, masseter, tongue, diaphragm gastrocnemius muscle, longissimus dorsi muscle and psoas major muscle) were collected at post-mortem for DNA extraction, pathology and mouse bioassay [16].

### Detection of porcine *T. gondii* IgG by enzyme linked immunosorbent assay (ELISA)

The ID Screen Toxoplasmosis Indirect Multi-specie ELISA kit (ID.vet, Montpellier, France) was used to examine IgG against *T. gondii* in porcine serum following the manufacturer's instructions [16]. An ELISA was valid if the mean value of the positive control optical density (ODpc) was greater than 0.35 (ODpc > 0.35), and if the ratio of the mean OD values for the positive and negative controls (ODpc and ODnc) were greater than 3 (ODpc/ODnc > 3). The interpretation of the result was presented as percent seropositivity (SP). A sample with an SP value of 50% or higher was considered positive, a negative result was an SP of 40% or less, and the result was classed as doubtful if the SP was between 40% and 50%.

### Histopathologic examination

Tissue (liver, kidney, lung, hilar lymph nodes, spleen, brain) samples from pigs were fixed in 10% neutral buffered formalin (NBF) at room temperature for 24 h. Following fixation, tissue samples were dehydrated through a graded series of ethanol (70%, 80%, 90%, and 100%), with each step lasting 30 min. Subsequently, they were cleared in xylene (two changes, 30 min each) and infiltrated with molten paraffin in a 58–60 °C oven for 1–2 h. The embedded tissues were then sectioned at 4–6 µm using a microtome. Finally, the sections were stained with hematoxylin and eosin (H&E) for microscopic evaluation of histopathological alterations [36].

### The microscopic examination and enumeration of *T. gondii* cysts in porcine brain tissue

The brain tissue from the G1, G3, and G5 groups was weighed, and 20 g of uniformly sampled brain tissue was placed in a mortar and homogenized to a paste-like consistency. A volume of 30 mL PBS was added to resuspend the homogenate. The brain homogenate was first transferred to a 15 mL centrifuge tube, followed by the careful addition of an equal volume of Ficoll-Paque^™^ PREMIUM (1.084) (Merck KGaA, GE17-5446-02, Germany) density gradient medium along the tube wall. Centrifugation was performed at 1,750 × *g* for 30 min. The bottom layer, which contained the *T. gondii* cysts, was gently aspirated. Microscopic examination and enumeration were conducted to calculate the cyst burden in each porcine brain sample [37].

### Detection of *T. gondii* B1 gene by reverse transcription quantitative polymerase chain reaction (RT-qPCR)

The genomic DNA of tissues (including brain, heart, gastrocnemius muscle, longissimus dorsi muscle, psoas major muscle and diaphragm) and *T. gondii* tachyzoites were extracted using the TIANamp Genomic DNA Kit (Tiangen Biotech, DP304-03, Beijing, China). DNA samples dissolved in distilled water were used to quantify the *T. gondii* DNA by amplifying the highly conserved B1 gene via RT-qPCR [38]. RT-qPCR was performed using forward primer F5′-AGGGACAGAAGTCGAAGGGG-3′ and reverse primer R5′-GCAGCCAAGCCGGAAACATC-3′ [38], amplifying a 164—bp fragment of B1 gene with SYBR Premix Ex *Taq* (Takara, Dalian, China). The following amplification protocol was applied: 10 min at 95 °C, 40 cycles at 95 °C for 15 s (denaturation), 62 °C for 45 s (annealing), and 72 °C for 30 s (amplification). Sample DNA underwent normalization utilizing the *T. gondii* B1 gene using the SYBR Premix Ex *Taq* to derive the CT values. Subsequently, parasite load was determined using the standard curve analysis based on the Lg (tachyzoite number) and the corresponding CT values of different gradient tachyzoites.

### Mouse bioassay

Tissues from pigs in G1, G3 and G5 for mouse bioassay were grouped differently. Based on the tissue type, collected tissues were divided into the following groups: “Brain” (50 g of brain), “Food” (a 50 g pooled sample which included 12.5 g each of chop, loin, left tricep and left semitendinosus), and “Other” (a 50 g pooled sample which included 12.5 g each of diaphragm, heart, tongue and masseter) [16]. These tissues were digested with acid/pepsin using a method previously described [39]. After the tissue homogenate was centrifuged for 10 min at 1,200 × *g*, the supernatant was poured off gently and the final pellet was resuspended in 3 mL sterile saline (which contained 400 µg/mL penicillin and 400 units/mL streptomycin). Six mice were intraperitoneally injected with 400 µL of each inoculum.

Mice surviving to the end of the 6-week bioassay were euthanized. Brain was stored separately in a sterile vial containing 1 ml PBS for DNA extraction. DNA extraction followed by PCR was conducted for all surviving mice used in the bioassay. The presence of *T. gondii* in mice brain was determined by PCR targeting *T. gondii* B1 gene as previously described [31].

### Statistical analysis

Experimental data were obtained for three biological replicates and analyzed using GraphPad Prism 8.0 (GraphPad Software Inc, CA, USA). A two-tailed, unpaired Student’s *t*-test was used to examine the significance of differences between two groups, including antibody levels and parasite burden. The Mantel-Cox log-rank test is employed to determine differences in survival curves. *P* < 0.05 was considered statistically significant, and *P* < 0.01, < 0.001, < 0.0001 represented varying degrees of significance.

## Results

### Protective efficacy of *T. gondii* knockout strains in pigs assessed by clinical responses post-immunization and post-challenge

**Fig. 2:**
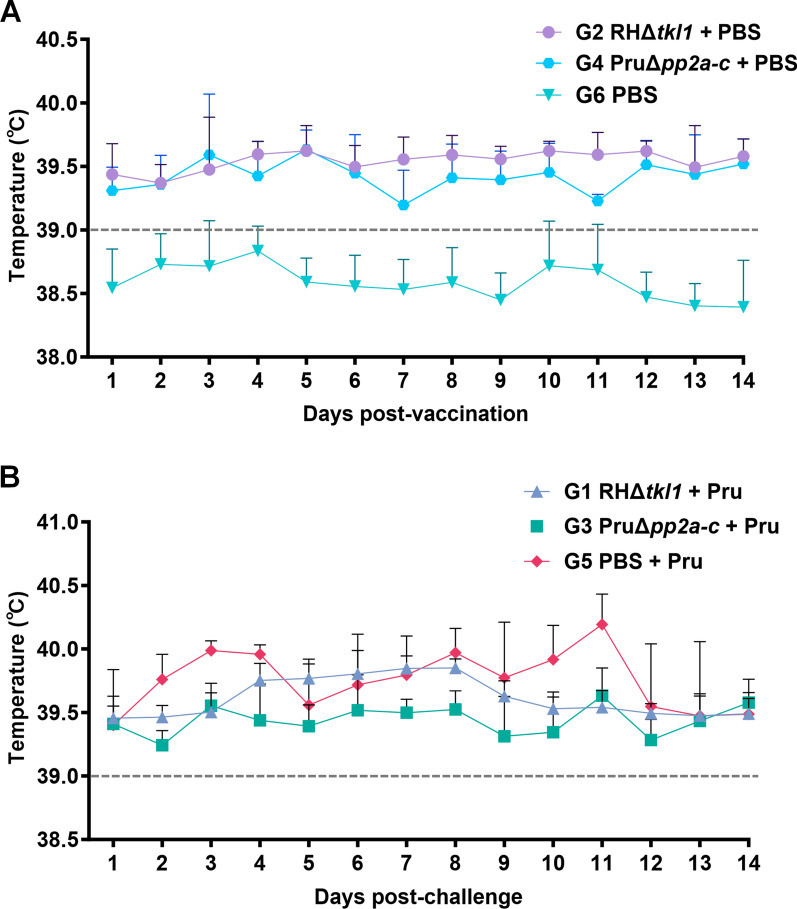
Mean body temperature of pigs in each experimental group post-vaccination and post-challenge. The gray cut off line indicates the normal rectal temperature for pigs. **A** Average porcine temperature per experimental group following vaccination with RHΔ*tkl1* or PruΔ*pp2a-c* tachyzoites. **B** Average porcine temperature per experimental group following challenge with oocysts of *Toxoplasma gondii* Pru strain

Following immunization with tachyzoites of *T. gondii* gene-knockout attenuated strains RHΔ*tkl1* and PruΔ*pp2a-c*, pigs exhibited mild anorexia and lethargy for two days, followed by a persistent low-grade fever (body temperature exceeding 39 °C) that persisted throughout the 14-day observation period, whereas the control group remained clinically normal, with good mental status, normal feed intake, and body temperatures not exceeding 39 °C (Fig. 2A). The RHΔ*tkl1* and PruΔ*pp2a-c* knockout strains of *T. gondii* were generally safe and well-tolerated in pigs, being associated only with transient and mild adverse effects. Following challenge with *T. gondii* Pru strain oocysts, pigs in groups G1, G3, and G5 all developed febrile responses characterized by rectal temperatures exceeding 39 °C within 2 weeks post-challenge, with the highest temperature reaching above 40 °C (Fig. 2B).

### Robust humoral response was elicited by vaccination with the RHΔ*tkl1* and PruΔ*pp2a-c* strains in pigs

**Fig. 3:**
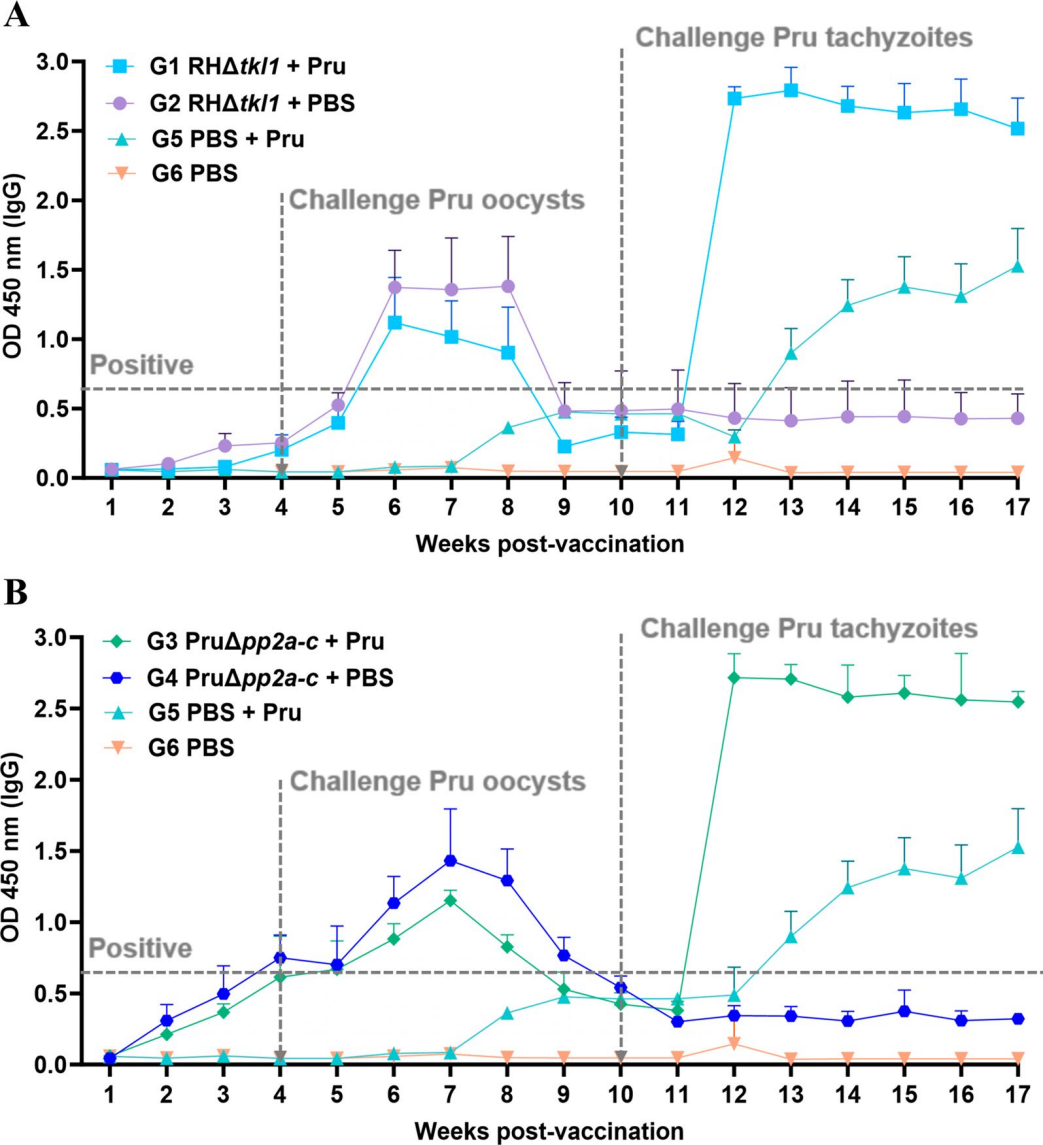
IgG levels in pig serum during immunization and *Toxoplasma gondii* challenge. ELISA was used to detect the *T. gondii*-specific IgG antibody levels in pigs at 1–11 weeks post-vaccination with RHΔ*tkl1* or PruΔ*pp2a-c* tachyzoites. The vertical dashed line with an arrow indicates the point at which pigs were challenged with 1,000 Pru *T. gondii* oocysts. The horizontal dashed line indicates when animals were classed as seropositive for *T. gondii*. **A** *T. gondii*-specific IgG antibody levels per experimental group in pigs vaccinated with RHΔ*tkl1* tachyzoites. **B** *T. gondii*-specific IgG antibody levels per experimental group vaccinated with PruΔ*pp2a-c* tachyzoites

At the beginning of the experiment, all pigs were tested negative for *T. gondii* IgG by ELISA. Following immunization with tachyzoites of the RHΔ*tkl1* strain, serum anti-*T. gondii* IgG antibodies in pigs from G1 and G2 increased slowly and remained negative throughout the initial 4-week period (Fig. 3A). After challenge with oocysts of the *T. gondii* Pru strain at week 4 post-vaccination, the *T. gondii*-specific IgG levels in G1 rose sharply to positive, peaked at week 6 and maintained this high level for 2 weeks, followed by a sharp decline and seroconversion to negative at week 9 (Fig. 3A). After booster challenge with tachyzoites of the *T. gondii* Pru strain at week 10 post-vaccination, the *T. gondii*-specific IgG levels in G1 rose to extremely high levels at week 12 and maintained this high level through week 17 (Fig. 3A). In contrast, G5 exhibited an upward trend in antibody levels beginning after week 7. After booster challenge with tachyzoites of the *T. gondii* Pru strain at week 10, the antibody levels rose and turned positive at week 13, continued to increase through week 15, and were still rising at week 17 (Fig. 3A). The blank control G6 consistently maintained very low and stable antibody levels throughout the observation period (Fig. 3A). Following immunization with tachyzoites of the PruΔ*pp2a-c* strain, serum anti-*T. gondii* IgG antibodies in pigs from G3 and G4 increased and tested positive by the fourth week (Fig. 3B). After challenge with the Pru oocysts at week 4 post-vaccination, the *T. gondii*-specific IgG levels in G3 continued to rise, peaked at week 7, and then declined from week 7 to week 11 (Fig. 3B). After booster challenge with tachyzoites of the *T. gondii* Pru strain at week 10 post-immunization, the *T. gondii*-specific IgG levels in G3 rose to extremely high levels at week 12 and maintained this high level through week 17 (Fig. 3B). It is worth noting that after the secondary challenge with *T. gondii* Pru tachyzoites, the peak levels of *T. gondii*-specific IgG antibodies in groups G1 and G3 were both significantly higher than those in group G5 (*P* < 0.01) (Fig. 3).

### Pathological changes in pig tissues

**Fig. 4:**
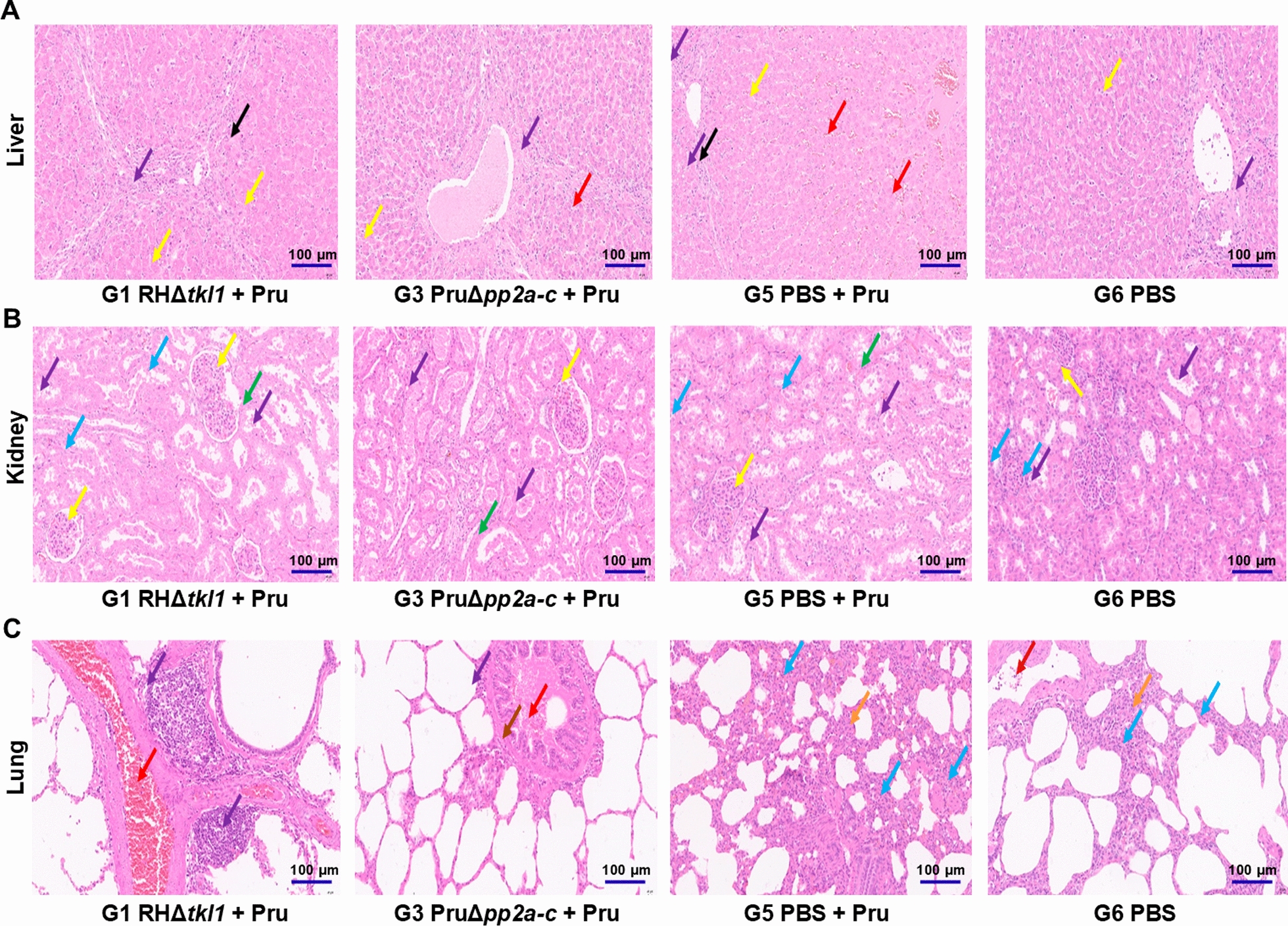
Histopathological results of pig organs and tissues. The organs displayed (liver, kidney, and lung) constitute a standard panel for assessing systemic health and pathological changes in experimental pigs. **A** Histopathological changes in liver tissue: mild steatosis is indicated by the yellow arrow; inflammatory cell infiltration is shown by the purple arrow; sinusoidal congestion is marked by the red arrow; and hepatocyte necrosis is denoted by the black arrow. **B** Histopathological changes in renal tissue: the yellow arrow indicates eosinophilic material within Bowman's capsule; the blue arrow points to hydropic degeneration of renal tubular epithelial cells; the purple arrow shows eosinophilic material and sloughed epithelial cells within the renal tubules; the black arrow denotes mild dilation of the renal tubules. **C** Histopathological changes in pulmonary tissue: the purple arrow indicates inflammatory cell infiltration; the blue arrow points to scattered granulocyte infiltration within the alveolar wall; the orange arrow shows alveolar wall thickening and widening of the interalveolar septa; the red arrow denotes interstitial vascular congestion; the brown arrow marks degeneration of bronchiolar epithelial cells

In the G5 group, hepatic tissues exhibited hepatocellular necrosis with focal sinusoidal congestion (Fig. 4A); multiple renal tubules showed mild dilation containing eosinophilic casts and sloughed epithelial cells (Fig. 4B); alveolar emphysema was observed with granulocyte infiltration in alveolar walls (Fig. 4C); the medulla of hilar lymph nodes demonstrated extensive lymphocyte depletion (Additional file 1: Figure S1A); splenic tissues presented vascular smooth muscle degeneration and sinusoidal dilation (Additional file 1: Figure S1B); cerebral meninges and parenchyma showed mild vascular congestion with numerous neurons displaying pyknotic and hyperchromatic nuclei (Additional file 1: Figure S1C). Histopathological lesions were attenuated in G1 and G3 groups. The histopathological scores of the liver, kidney, lung, hilar lymph nodes, spleen, and brain tissue in pigs from groups G1, G3, G5, and G6 are shown in Additional file 2: Table S1-S6.

### Significant reduction in brain cyst formation following immunization with RHΔ*tkl1* or PruΔ*pp2a-c*

**Fig. 5:**
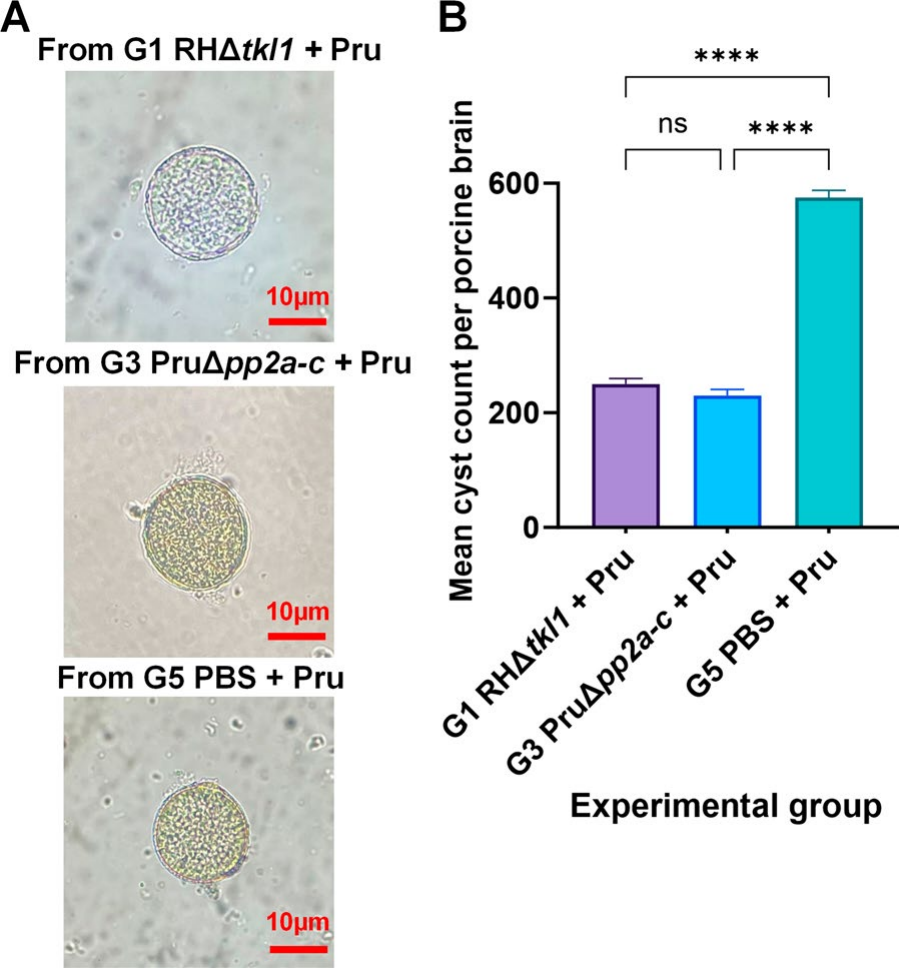
Brain cysts in pig brain tissues across groups and statistical analysis of cyst counts. **A** Brain cysts from pigs originating from G1, G3, and G5, respectively. **B** The mean number of brain cysts in pigs was significantly reduced in G1 (vaccinated with RHΔ*tkl1* tachyzoites and challenged) and G3 (vaccinated with PruΔ*pp2a-c* tachyzoites and challenged) compared to group G5 (non-vaccinated but challenged) (****P < 0.0001)

Microscopic examination of brain tissues from pigs in groups G1, G3, and G5 revealed the presence of tissue cysts (Fig. 5A). The mean brain cyst burden was quantified as 250 ± 10 in G1, 230 ± 11 in G3, and 575 ± 13 in G5. Compared to the G5 group without immunization, pigs in groups G1 and G3 exhibited a significant reduction in the mean number of brain cysts (*P* < 0.0001) (Fig. 5B).

### Effective reduction of tissue *T. gondii* load in pigs immunized with RHΔ*tkl1* or PruΔ*pp2a-c*

**Fig. 6:**
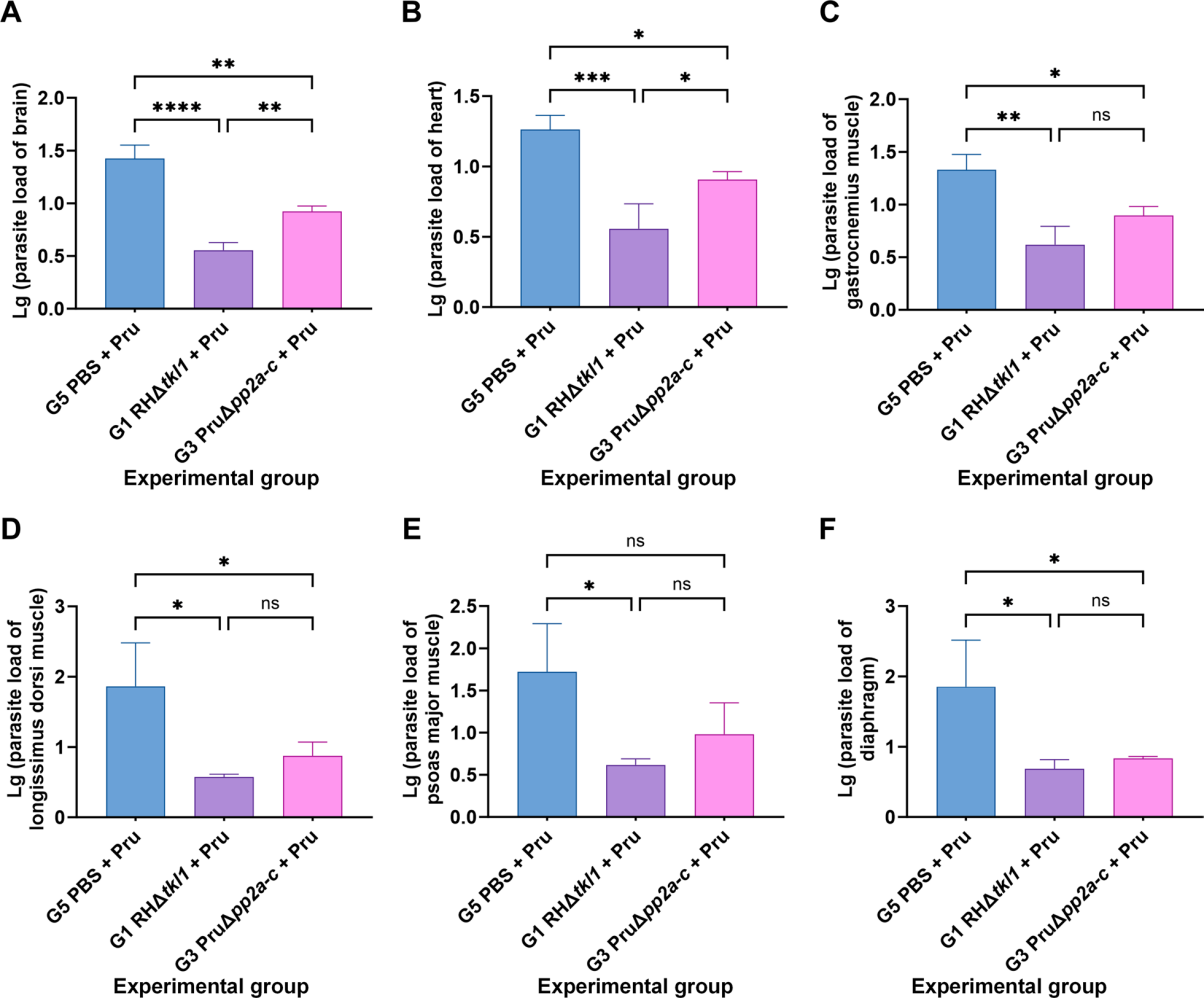
Parasite burden in the brain (**A**), heart (**B**), gastrocnemius muscle (**C**), longissimus dorsi muscle (**D**), psoas major muscle (**E**) and diaphragm (**F**) of pigs immunized with 1 × 10^7^ RHΔ*tkl1* or PruΔ*pp2a-c* tachyzoites at day 0, which were assessed by reverse transcription quantitative polymerase chain reaction quantitative PCR at days 120 post-vaccination. ****P < 0.0001, ***P < 0.001, **P < 0.01, *P < 0.05

In brain tissue, compared with the unimmunized G5 group, the *T. gondii* load in the G1 and G3 groups was significantly reduced (*P* < 0.01), and the *T. gondii* load in the G1 group was significantly lower than that in the G3 group using RT-qPCR (*P* < 0.01) (Fig. 6A). In heart tissue, the *T. gondii* load in the G1 and G3 groups was significantly decreased compared with the unimmunized G5 group (*P* < 0.05), with the G1 group showing a significantly lower load than the G3 group (*P* < 0.05) (Fig. 6B). In gastrocnemius muscle tissue, the *T. gondii* load in the G1 and G3 groups was significantly reduced relative to the unimmunized G5 group (*P* < 0.05) (Fig. 6C). In longissimus dorsi muscle tissue, the *T. gondii* load in the G1 and G3 groups was significantly lower than that in the unimmunized G5 group (*P* < 0.05) (Fig. 6D). In psoas major muscle tissue, the *T. gondii* load in the G1 group was significantly reduced compared with the G5 group (*P* < 0.05) (Fig. 6E). In diaphragm tissue, the *T. gondii* load in the G1 and G3 groups was significantly decreased relative to the unimmunized G5 group (*P* < 0.05) (Fig. 6F).

### Detection of *T. gondii* DNA from mouse brains following bioassay

All mice inoculated with tissue digesta survived until the end of the 6-week experiment. *T. gondii* DNA was detected in the brain tissues of 68.1% (49/72, 95% *CI* 57.3–78.8) of mice inoculated with tissue from pigs in G5 (Table 2); whereas *T. gondii* DNA was detected in only 12.5% (9/72, 95% *CI* 4.9–20.1) of mice inoculated with G1 swine tissue and 19.4% (14/72, 95% *CI* 10.3–28.6) of those inoculated with G3 swine tissue, significantly lower than that from group G5 (*P* < 0.0001) (Table 2).

**Table 2:** Prevalence and variation of *Toxoplasma gondii* DNA in mouse brains post-inoculation with pig tissue digestates among groups

Group	No. Tested	No. Positives	Prevalence % (95% *CI*)	Odds ratio (95% *CI*)	*P*-value
G1	72	“Brain” group 6/24	12.5 (4.9–20.1)	1	< 0.001
		“Food” group 1/24			
		“Other” group 2/24			
G3	72	“Brain” group 6/24	19.4 (10.3–28.6)	1.7 (0.7–4.2)	
		“Food” group 5/24			
		“Other” group 3/24			
G5	72	“Brain” group 23/24	68.1 (57.3–78.8)	14.9 (6.3–35.1)	
		“Food” group 20/24			
		“Other” group 6/24			

## Discussion

Pork represents one of the most significant sources of human *T. gondii* infection, underscoring the critical need for a porcine-specific toxoplasmosis vaccine focused on reducing tissue cyst burden [40]. The present study showed that vaccination with live attenuated *T. gondii* strains (RHΔ*tkl1* or PruΔ*pp2a-c*) can successfully and significantly reduce infective tissue cysts in tissues of the immunized pigs. Furthermore, the mouse bioassay included inoculations from three different tissue categories—“food” tissue group (chop, loin, left triceps and left semitendinosus), “brain” and “other” tissue groups (diaphragm, heart, tongue and masseter)—based on the fact that alimentary tissues are most commonly consumed by humans. Mouse bioassay has been the “gold” standard for assessing the viability of *T. gondii* tissue cysts [39, 41]. In previous studies on swine vaccination for reducing tissue cysts, the mouse bioassay constituted an extremely critical research component. These studies have primarily focused on the microscopic identification of tissue cysts and the detection of parasitic DNA in mice used for bioassays [8, 17, 42–44]. In a study by Burrells et al., mouse bioassay combined with molecular detection of *T. gondii* DNA was employed to evaluate the efficacy of vaccination using the incomplete S48 strain in reducing tissue cyst burden in pigs following challenge with M4 strain oocysts. The bioassay results revealed that all mice receiving porcine tissues from vaccinated and challenged pigs survived, whereas only 51.1% of mice that received tissues from non-vaccinated, challenged pigs survived [16]. In our study, the results of the mouse bioassay showed that the proportion of *T. gondii* DNA in the brain tissues of mice that received porcine tissues from pigs immunized with RHΔ*tkl1* or PruΔ*pp2a-c* and subsequently challenged was significantly reduced compared with that in mice that received tissues from non-immunized but challenged pigs (*P* < 0.001). A study by Burrells et al. showed that the main predilection sites for *T. gondii* in pigs were the brain and highly vascular muscles (such as tongue, diaphragm, heart and masseter), whereas the meat cuts (such as chop, loin, left tricep and left semitendinosus) displayed a lower burden of *T. gondii* tissue cysts [16]. In our study, the results of the mouse bioassay further confirmed this finding, with the highest proportion of *T. gondii* DNA detected in the brain tissues of mice that received porcine brain tissues from non-immunized but challenged pigs. In this study, *T. gondii*-specific IgG antibodies in pigs did not reach positive levels following challenge with Pru oocysts, reflecting that the efficacy of the Pru oocyst challenge was suboptimal. Therefore, the results of the mouse bioassay were based on a secondary challenge of pigs with *T. gondii* Pru tachyzoites.

Clinical symptoms observed after *T. gondii* infection in pigs varied according to the breed and age of the pigs, the number and developmental stage of *T. gondii* administrated, and the administration route of *T. gondii* [45]. Following *T. gondii* infection, pigs manifest clinical signs such as fever, anorexia, and a generally poor condition [46]. In the present study, pigs challenged with *T. gondii* Pru oocysts exhibited high fever with rectal temperatures reaching above 40 °C, whereas pigs inoculated with attenuated RHΔ*tkl1* or PruΔ*pp2a-c* strains developed a low-grade fever, accompanied by transient anorexia and lethargy that resolved shortly thereafter. Moreover, based on histopathological changes and pathological scores, compared with the non-immunized group challenged with *T. gondii* Pru oocysts and tachyzoites, the group that was immunized and subsequently challenged with *T. gondii* Pru oocysts and tachyzoites showed significantly alleviated pathological changes in certain tissues. Therefore, the attenuated RHΔ*tkl1* and PruΔ*pp2a-c* strains exhibit a certain degree of safety and tolerability in swine.

Burrells et al. challenged pigs with oocysts, and 2 weeks post challenge, pigs were *T. gondii* IgG positive [16]. In our study, following challenge infection with *T. gondii* Pru oocysts, the *T. gondii*-specific IgG antibodies in G5 pigs remained negative throughout the subsequent 6-week period. This indicates that the infection outcome was suboptimal, which we consider may be attributed to factors such as oocyst virulence, experimental procedures, or individual variation. Subsequently, at week 10 of the experiment, we performed a secondary challenge infection with *T. gondii* Pru tachyzoites. By the third week following the secondary challenge, *T. gondii*-specific antibodies turned positive. Following vaccination with RHΔ*tkl1* and subsequent challenge with *T. gondii* Pru oocysts at week 4, *Toxoplasma*-specific IgG antibodies continuously increased, peaking at week 6. In contrast, vaccination with PruΔ*pp2a-c* led to a continuous rise in IgG levels that peaked at week 7, indicating a more prolonged antibody response. In G1 and G3 groups, following secondary challenge infection with *T. gondii* Pru tachyzoites, the *T. gondii*-specific antibodies exhibited a consistently upward trend and remained at high levels until week 17. Notably, although the *T. gondii*-specific antibody IgG levels in pigs vaccinated with RHΔ*tkl1* showed an upward trend, the antibody levels remained consistently negative until week 4 of the experiment. Pigs develop good protective immunity as evidenced by recovery from infection with viable *T. gondii* [47]. This is associated with the biological characteristics of the parasite strain, functional differences in the knocked-out genes, and the immune response profile of pigs [47]. *T. gondii* type I strain RH exhibits rapid proliferation but limited survival in pigs, resulting in poor tissue cyst formation and transient antigenic stimulation, which likely contributes to a delayed antibody response [47, 48]. In addition, the RHΔ*tkl1* strain tends to induce a Th1 type immune response, characterized by a rapid and robust cellular immune reaction, whereas the humoral immune response may be relatively delayed [35].

Though previous studies on vaccination of pigs against *T. gondii* have not documented the microscopic observation of tissue cysts [47, 49], in the present study, tissue cysts were observed in porcine brains. Moreover, the immunized groups exhibited a significant reduction in brain cyst counts compared to the challenge control group (*P* < 0.0001). This result provides direct evidence that vaccination effectively reduces tissue cyst formation in pigs.

Quantification of *T. gondii* load in various pig tissues via RT-qPCR targeting the *T. gondii* B1 gene showed that the *T. gondii* load in G1 and G3 immunized groups was significantly lower than that in the unimmunized G5 group across most porcine tissues. Specifically, in brain tissue (*P* < 0.01) and heart tissue (*P* < 0.05), G1 exhibited a further significant reduction in parasite load compared to G3. For gastrocnemius muscle, longissimus dorsi muscle and diaphragm, both G1 and G3 groups displayed marked decreases in *T. gondii* load relative to G5 (*P* < 0.05). Notably, only G1 showed a significant reduction of *T. gondii* load in psoas major muscle compared with G5 (*P* < 0.05). RT-qPCR results demonstrated that the attenuated RHΔ*tkl1* strain exhibited better efficacy than the PruΔ*pp2a-c* strain. Both RHΔ*tkl1* and *Pru*Δ*pp2a-c* strains have demonstrated significant efficacy in reducing the formation of tissue cysts in pigs. Comparative evaluation via mouse bioassay and tissue cyst quantification indicates that the RHΔ*tkl1* strain induces a more marked reduction in *T. gondii* tissue burden in pigs than the PruΔ*pp2a-c* strain.

This study has several limitations. First, one limitation of the experimental design was that following the primary challenge with *T. gondii* Pru oocysts, the intended infection outcome was not achieved, as reflected by the *T. gondii*-specific antibody IgG responses. Consequently, a secondary challenge with *T. gondii* Pru tachyzoites was subsequently performed. Second, the humoral and cellular immune responses in pigs following vaccination with the attenuated *T. gondii* strains were assessed using a limited set of indicators and parameters; a broader and more comprehensive panel of indicators and parameters would have allowed for a more robust assessment of the immune effects of the candidate vaccine strains. Research on vaccines against porcine toxoplasmosis faces numerous challenges, including the complex life cycle of *T. gondii*, its immune evasion mechanisms, strain diversity, adjuvant selection, and co-infection with other pathogens. An ideal vaccine against porcine toxoplasmosis should possess absolute safety, high efficacy, and induce durable immune responses.

## Conclusions

Our study shows that the two live attenuated RHΔ*tkl1* and PruΔ*pp2a-c* strains of *T. gondii* are relatively safe, immunogenic, and confer significant protection in pigs against challenge with both oocysts and tachyzoites of the Pru strain. The data on antibody levels, pathological changes, pathological score, parasite load, brain cyst counts, and mouse bioassay collectively demonstrate that the two live attenuated *T. gondii* strains are potential candidate vaccines against porcine toxoplasmosis.

## Supplementary Information

Additional file 1: Figure S1. Histopathological results of pig organs and tissues. The organs displayed (hilar lymph nodes, spleen, and brain) constitute a standard panel for assessing systemic health and pathological changes in experimental pigs. A Histopathological changes in the hilar lymph nodes tissue: the red arrow indicates a lymphoid nodule; the black arrow points to mild dilation of the medullary sinus; the blue arrow shows a decreased lymphocyte density in a relatively extensive area of the medulla, with loosely arranged connective tissue. B Histopathological changes in splenic tissue: the blue arrow indicates scattered granulocyte infiltration; the yellow arrow points to degeneration of vascular smooth muscle cells. C Histopathological changes in brain tissue: the black arrow indicates pyknosis and hyperchromasia of neuronal nuclei; the red arrow points to vascular congestion; the purple arrow shows scattered lymphocyte infiltration.

Additional file 2: Table S1. Histopathological scores of porcine livers in the RHΔ*tkl1 *+ Pru group, PruΔ*pp2a-c *+ Pru group, PBS + Pru group, and PBS group. Table S2. Histopathological scores of porcine kidneys in the RHΔ*tkl1 *+ Pru group, PruΔ*pp2a-c *+ Pru group, PBS + Pru group, and PBS group. Table S3. Histopathological scores of porcine lungs in the RHΔ*tkl1 *+ Pru group, PruΔ*pp2a-c *+ Pru group, PBS + Pru group, and PBS group. Table S4. Histopathological scores of porcine hilar lymph nodes in the RHΔ*tkl1* + Pru group, PruΔ*pp2a-c* + Pru group, PBS + Pru group, and PBS group. Table S5. Histopathological scores of porcine spleens in the RHΔ*tkl1* + Pru group, PruΔ*pp2a-c* + Pru group, PBS + Pru group, and PBS group. Table S6. Histopathological scores of porcine brains in the RHΔ*tkl1* + Pru group, PruΔ*pp2a-c* + Pru group, PBS + Pru group, and PBS group.

## Data Availability

The datasets supporting the findings of this article are provided within the paper and supplementary materials.

## Abbreviations

ELISA: Enzyme-linked immunosorbent assay
CRISPR: Clustered regularly interspaced short palindromic repeats
*tkl1*: Tyrosine kinase-like 1
PP2A: Protein phosphatase 2A
HFF: Human foreskin fibroblast
DMEM: Dulbecco’s Modified Eagle medium
FBS: Fetal bovine serum
NBF: Neutral buffered formalin
IM: Intramuscular
H&E: Hematoxylin and eosin
RE: Repetitive
PCR: Polymerase chain reaction
RT-qPCR: Reverse transcription quantitative polymerase chain reaction
