# Supplementary figures and images for: Live attenuated RHΔtkl1 and PruΔpp2a-c mutants of Toxoplasma gondii are promising vaccine candidates conferring protection in pigs
Source: Infect Dis Poverty. 2026 May 6;15:49. doi: 10.1186/s40249-026-01451-8 (PMC13147800; doi:10.1186/s40249-026-01451-8)

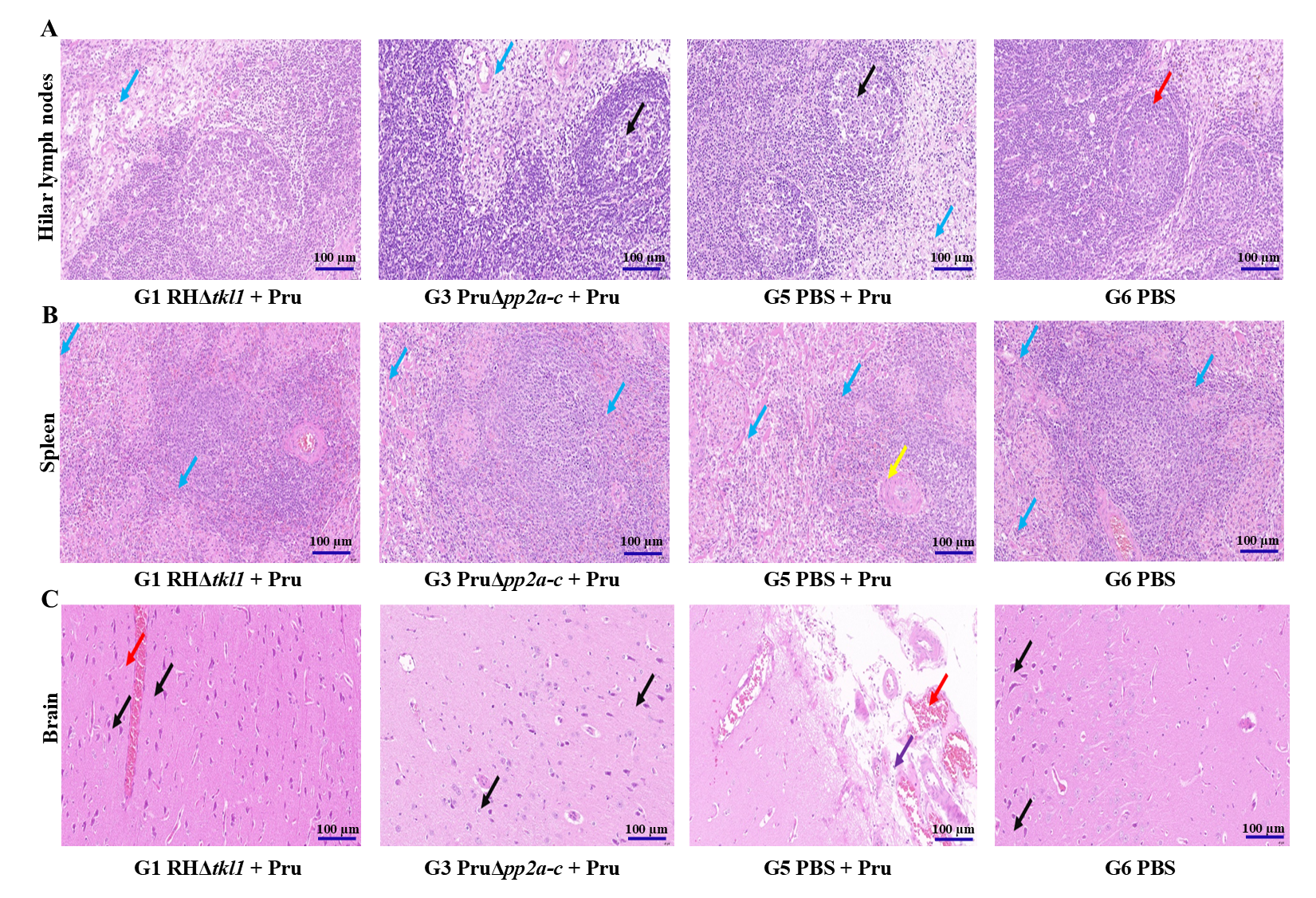

Supplement: Supplementary file 1 — Additional file 1: Figure S1. Histopathological results of pig organs and tissues. The organs displayed (hilar lymph nodes, spleen, and brain) constitute a standard panel for assessing systemic health and pathological changes in experimental pigs. A Histopathological changes in the hilar lymph nodes tissue: the red arrow indicates a lymphoid nodule; the black arrow points to mild dilation of the medullary sinus; the blue arrow shows a decreased lymphocyte density in a relatively extensive area of the medulla, with loosely arranged connective tissue. B Histopathological changes in splenic tissue: the blue arrow indicates scattered granulocyte infiltration; the yellow arrow points to degeneration of vascular smooth muscle cells. C Histopathological changes in brain tissue: the black arrow indicates pyknosis and hyperchromasia of neuronal nuclei; the red arrow points to vascular congestion; the purple arrow shows scattered lymphocyte infiltration. [file 40249_2026_1451_MOESM1_ESM.tif]
