# Supplementary material for: Live attenuated RHΔtkl1 and PruΔpp2a-c mutants of Toxoplasma gondii are promising vaccine candidates conferring protection in pigs
Source: Infect Dis Poverty. 2026 May 6;15:49. doi: 10.1186/s40249-026-01451-8 (PMC13147800; doi:10.1186/s40249-026-01451-8)
Supplement: Supplementary file 2 — Additional file 2: Table S1. Histopathological scores of porcine livers in the RHΔtkl1 + Pru group, PruΔpp2a-c + Pru group, PBS + Pru group, and PBS group. Table S2. Histopathological scores of porcine kidneys in the RHΔtkl1 + Pru group, PruΔpp2a-c + Pru group, PBS + Pru group, and PBS group. Table S3. Histopathological scores of porcine lungs in the RHΔtkl1 + Pru group, PruΔpp2a-c + Pru group, PBS + Pru group, and PBS group. Table S4. Histopathological scores of porcine hilar lymph nodes in the RHΔtkl1 + Pru group, PruΔpp2a-c + Pru group, PBS + Pru group, and PBS group. Table S5. Histopathological scores of porcine spleens in the RHΔtkl1 + Pru group, PruΔpp2a-c + Pru group, PBS + Pru group, and PBS group. Table S6. Histopathological scores of porcine brains in the RHΔtkl1 + Pru group, PruΔpp2a-c + Pru group, PBS + Pru group, and PBS group. [file 40249_2026_1451_MOESM2_ESM.pdf]

Additional file 2: Table S1. Histopathological scores of porcine livers in the RHΔ*tkl1* + Pru group, PruΔ*pp2a-c* + Pru group, PBS + Pru group, and PBS group.

Group	Necrosis	Hepatic steatosis	Congestion	Inflammatory cell infiltration
RHΔ*tkl1* + Pru	0	0	0	0
PruΔ*pp2a-c* + Pru	0	0	0	0
PBS + Pru	0	1	1	1
PBS	1	0	0	0

Note: 0 = within normal range, 1 = very mild, 2 = mild, 3 = moderate, 4 = severe.

Additional file 2: Table S2. Histopathological scores of porcine kidneys in the RHΔ*tkl1* + Pru group, PruΔ*pp2a-c* + Pru group, PBS + Pru group, and PBS group.

Group	Tubular epithelial cell degeneration	Tubular epithelial cell desquamation	Tubular epithelial cell necrosis	Tubular dilatation
RHΔ*tkl1* + Pru	1	0	0	0
PruΔ*pp2a-c* + Pru	1	1	0	1
PBS + Pru	1	2	0	2
PBS	1	1	0	1

Note: 0 = within normal range, 1 = very mild, 2 = mild, 3 = moderate, 4 = severe.

Additional file 2: Table S3. Histopathological scores of porcine lungs in the RHΔ*tkl1* + Pru group, PruΔ*pp2a-c* + Pru group, PBS + Pru group, and PBS group.

Group	Inflammatory cell infiltration	Alveolar wall thickening	Alveolar dilatation	Hemorrhage
RHΔ*tkl1* + Pru	1	1	1	0
PruΔ*pp2a-c* + Pru	0	0	0	1
PBS + Pru	2	2	1	0
PBS	2	0	0	0

Note: 0 = within normal range, 1 = very mild, 2 = mild, 3 = moderate, 4 = severe.

Additional file 2: Table S4. Histopathological scores of porcine hilar lymph nodes in the RHΔ*tkl1* + Pru group, PruΔ*pp2a-c* + Pru group, PBS + Pru group, and PBS group.

Group	Necrosis	Inflammatory cell infiltration	Congestion
RHΔ*tkl1* + Pru	0	0	0
PruΔ*pp2a-c* + Pru	0	0	0
PBS + Pru	0	1	1
PBS	0	0	0

Note: 0 = within normal range, 1 = very mild, 2 = mild, 3 = moderate, 4 = severe.

Additional file 2: Table S5. Histopathological scores of porcine spleens in the RHΔ*tkl1* + Pru group, PruΔ*pp2a-c* + Pru group, PBS + Pru group, and PBS group.

Group	Necrosis	Inflammatory cell infiltration	Hemorrhage
RHΔ*tkl1* + Pru	0	0	0
PruΔ*pp2a-c* + Pru	0	0	0
PBS + Pru	0	0	1
PBS	0	0	0

Note: 0 = within normal range, 1 = very mild, 2 = mild, 3 = moderate, 4 = severe.

Additional file 2: Table S6. Histopathological scores of porcine brains in the RHΔ*tkl1* + Pru group, PruΔ*pp2a-c* + Pru group, PBS + Pru group, and PBS group.

Group	Necrosis	Inflammatory cell infiltration	Congestion
RHΔ*tkl1* + Pru	0	0	0
PruΔ*pp2a-c* + Pru	0	0	0
PBS + Pru	0	1	1
PBS	0	0	0

Note: 0 = within normal range, 1 = very mild, 2 = mild, 3 = moderate, 4 = severe.
